# Concentrations of 2,4-Dichlorophenol and 2,5-Dichlorophenol in Urine of Korean Adults

**DOI:** 10.3390/ijerph15040589

**Published:** 2018-03-25

**Authors:** Hyejin Park, Kisok Kim

**Affiliations:** 1 Department of International Healthcare Administration, Daegu Catholic University, Kyungbuk 38430, Korea; hjpark@cu.ac.kr; 2 College of Pharmacy, Keimyung University, Daegu 42601, Korea; kimkisok@kmu.ac.kr

**Keywords:** human urine, biomonitoring, chlorophenol, demographic characteristics

## Abstract

Humans are exposed to the environmental pollutants 2,4-dichlorophenol (2,4-DCP) and 2,5-dichlorophenol (2,5-DCP) through air, the use of water and the consumption of products. In this study, we evaluated the urinary concentrations of these compounds in Korean people between the ages of 18 to 69 years, by making use of data from the Korean National Human Biomonitoring Survey that was completed in 2009. Of 1865 representative Koreans, 63.4% and 97.9% were found to have concentrations of 2,4-DCP and 2,5-DCP > 0.05 μg/L (limit of detection) in their urine, respectively. The geometric mean of urinary concentrations was 0.14 μg/L (confidence interval of 95% = 0.13–0.16) and 0.44 μg/L (confidence interval = 0.41–0.48), respectively. It was found that the adjusted proportional changes in 2,4-DCP concentrations were significantly associated with body mass index, whereas those of 2,5-DCP concentrations were influenced by place of residence. From these findings, it is evident that most adults in Korea have levels of 2,4-DCP and 2,5-DCP that are detectable in their urine and the burden of these compounds on their bodies varies depending on numerous demographic factors.

## 1. Introduction

The presence of toxic chemicals in consumer products, water, and air pose a wide range of health risks to the general population. Therefore, biomonitoring studies of potentially toxic substances are crucial for establishing the level of exposure a population has to these substances, identifying groups that are at high risk, describing differences in geography, and evaluating the health risks that a population faces [1]. It is for these purposes that many countries, including the U.S. and Germany, have carried out national biomonitoring studies, including analyses of organochlorine compounds. 2,4-dichlorophenol (2,4-DCP) and 2,5-dichlorophenol (2,5-DCP) are the most common organochlorine compounds to be easily found in soils and waste streams [2,3].

2,4-DCP is a key component in the production of phenoxy acid herbicides and is also used in the synthesis of antiseptics and pharmaceuticals [3]. This substance can also find its way into the environment as a product of degradation of an antiseptic agent known as triclosan [4]. Furthermore, 2,5-DCP acts as a key metabolite of 1,4-dichlorobenzene (1,4-D), which forms from water and wastewater treatment, wood pulp processing, incineration processes, and the degradation/metabolism of 1,4-D. 2,5-DCP has also been applied as a chemical intermediate in the production of pharmaceutical and agricultural products and dyes. Both 2,4-DCP and 2,5-DCP in drinking and waste water are byproducts of water treatment by chlorination [3].

Exposure to chlorinated phenols induces toxic effects in many animals, as well as humans [5,6,7]. Epidemiological studies suggest that exposure to dichlorophenol increases morbidity due to asthma, total serum levels of immunoglobulin E (IgE) in patients with atopy and a history of wheezing, and contributes to the increasing incidence of food allergies [8,9]. Furthermore, exposure to 2,5-DCP causes metabolic syndrome, diabetes, and obesity, while concentrations of 2,4-DCP in urine are reportedly significantly associated with olfactory dysfunction [10,11,12,13,14]. Following exposure, 2,4-DCP and 2,5-DCP are readily absorbed from the gastrointestinal tract and metabolized to glucuronide and other conjugates. Then the parent compound and its conjugates are rapidly eliminated from the body, mainly through the urine. It is known that the half-life of 2,4-DCP and its conjugates in plasma, fat, brain, liver, and kidney ranges from 4 to 30 min [3].

According to previously-conducted biomonitoring surveys of phenolic compounds, urinary concentrations vary significantly depending on the population sub-groups. This finding reflects the difference in the burdens these compounds have on bodies, which vary according to geographical region [15,16]. In addition, epidemiological studies have demonstrated factors that are relevant to the burdens on the body after exposure to phenolic compounds. These factors include age, household income, and cigarette smoking [16,17]. In the current study, we used nation-wide biomonitoring data to estimate the concentrations of 2,4-DCP and 2,5-DCP in the urine of Korean adults. We also investigated the demographic factors that determine the concentrations of these substances.

## 2. Materials and Methods

### 2.1. Study Population

The data used in this study were obtained from the Korean National Human Biomonitoring Survey (KNHBS). The KNHBS surveys and gathers population-based information on the body burden of hazardous chemicals in the Korean adult population, between the ages of 18–69 years. The World Health Organization (WHO) recommends the exclusion of participants when samples are too diluted or too concentrated. According to WHO, creatinine concentrations of <30 mg/dL are considered too diluted, while creatinine concentrations of >300 mg/dL are considered too concentrated [18]. A total of 1865 participants completed interviews by providing the required data and produced urine samples that were used in this study. The review and approval of the study was conducted by the Asan Medical Center Institutional Review Board (approval # AMC IRB-2009-0369). The ethical principles for medical research involving human subjects were used to guide the study, as provided for by the Declaration of Helsinki. All participants provided informed written consent.

### 2.2. Source of Data

Data were acquired from participants through in-person interviews. The information collected, included: Age, sex, income, education level, cigarette smoking, and geographical place of residence. There were four groups in the income category with regard to monthly household income. The level of education had three classifications: Less than high school, high school, and college or above. Cigarette smoking was classified as never, current, and former. The urban/rural category was classified based on the national administrative units, primarily according to population size (urban ≥ 50,000). Body mass index (BMI) was categorized based on WHO’s criteria for Asian populations: underweight (BMI < 18.5 kg/m^2^), normal weight (BMI = 18.5–22.9 kg/m^2^), overweight (BMI = 23.0–25.0 kg/m^2^), or obese (BMI ≥ 25.0 kg/m^2^) [19]. Samples of spot urine were received from all participants throughout the day at the interview sites. Each participant was asked to fill the urine collection cup (120 mL). For each urine sample, a 5 mL aliquot of urine was placed into a sterile conical polypropylene tube (10 mL) and stored in a freezer at −20 °C prior to analysis. Using urine creatinine concentrations, urinary dilutions of the samples were adjusted. Analyses of these samples were performed via liquid–liquid extraction and gas chromatography-mass spectrometry (GC-MS). In brief, the urine samples were thawed at room temperature and vortex-mixed. After gentle mixing, the 2 mL of samples were spiked with 20 μL of 5 mg/mL 2,4-dichlorophenol-3,5,6-*d*_3_ as an internal standard. They were then added to 50 μL of glucuronidase/arylsulfatase solution (in 0.2 M sodium acetate buffer, pH 5.2) and hydrolysis proceeded at 55 °C for 3 h. After cooling to room temperature, the samples were added to a 5% K_2_CO_3_ solution and extracted with methyl tert-butyl ether. The dried extracts were derivatized with 50 μL of a bis-(trimethylsilyl) trifluoroacetamide/trimethylchlorosilane (100:1, *v*/*v*) mixture, at 60 °C for 30 min. The samples were analyzed using GC-MS. Details of this method have been described elsewhere [20]. To perform the recovery tests, known amounts of standard were added to the samples, and values of 89.9–105.0% were obtained for 2,4-DCP and 90.5–105.5% for 2,5-DCP. The inter-day and intra-day accuracy and precision were approximated by the analysis of four analytes in seven replicates on a single day, for five consecutive days. For 2,4-DCP, the intra-day accuracy was at about 98.6–108.2%, with a precision of 1.9–10.6%, while its inter-day accuracy was at about 97.2–103.0%, with a precision of 3.6–6.0%. The intraday accuracy of 2,5-DCP was 97.1–110.6%, with a precision of 2.9–10.0%, while the inter-day accuracy was about 97.5–104.2%, with a precision of 2.6–15.4%. Linearity was checked from limit of quantification (LOQ) of 0.1 to 200 μg/L, with correlation coefficients of 0.9982 for 2,4-DCP, and 0.9998 for 2,5-DCP. The limit of detection (LOD) and the LOQ for analytes within the chromatographic conditions were obtained at signal-to-noise ratios of 3 and 10, respectively. The LOD and LOQ for 2,4-DCP and 2,5-DCP were 0.05 μg/L and 0.10 μg/L, respectively. Participants whose concentration of urine was found to be below the LOD were assigned with a value equivalent to LOD/2 [21]. The levels of creatinine in the urine samples were measured by a kinetic Jaffé method that employs the use of a Hitachi 7600 auto-analyzer (Hitachi, Tokyo, Japan).

### 2.3. Statistical Analyses

The distribution levels of 2,4-DCP and 2,5-DCP were described using selected maximum values and percentiles. To determine geometric means for the concentrations of 2,4-DCP and 2,5-DCP in urine samples with confidence intervals (CIs) of 95%, the antilog values of the means were calculated from the natural log-transformed values. An adjustment was performed for unequal selection probabilities by using sample weights of participants to calculate weighted geometric means. Linear regressions of log-transformed concentrations were calculated using the sample weights in order to obtain predictor variables. The interpretation of the exponentiated model coefficients can be taken as proportional changes in the value of the mean that is associated with each predictor level relative to a reference level, making the required adjustments for other predictors within the model. A *t*-test was used to analyze the differences between two means obtained from two different groups, and to test for linearity among the groups and the significance of a linear contrast in the linear models. All statistical analyses were completed using SAS software, version 9.4 (SAS Institute, Cary, NC, USA).

## 3. Results

A total of 1865 subjects were selected to participate in the study with a participation rate of 87.1%. The average age of the participants was 45.5 years, with 57% comprising of women. Table 1 presents the percentiles of the levels of 2,4-DCP in the urine samples collected from participants. From these results, it is evident that 63.4% of the samples had levels of 2,4-DCP above the LOD—ranging from above 0.05 μg/L to 83.6 μg/L. Females had a slightly lower concentration compared to male participants at most percentile points. It is however, noted that the creatinine-adjusted levels were significantly higher for females compared to males at all percentile points. It is also noted that the concentration of subjects in the age bracket of 60–69 were lower compared to those of other age brackets at most of the percentile points. However, there was no significant difference in the creatinine adjusted concentrations for this particular age group at most percentile points in comparison to other age groups.

In Table 2, selected percentiles of levels of 2,5-DCP in the urine sample of participants are shown by sex and age. 2,5-DCP was detected to be above 0.05 μg/L (LOD) in 97.9% of participants. The total concentration fell in the range between 0.05 and 63.0 μg/L. Even though no apparent difference was noted between males and females, females recorded significantly higher creatinine-adjusted concentrations compared to males. There was no obvious relationship between the concentration and age.

From Table 3, the weighted geometric mean for concentrations of 2,4-DCP in the samples of urine at a confidence interval of 0.13–0.16 was 0.14 μg/L. There was no significant relationship between the concentrations of 2,4-DCP and the demographic factors studied. There was a significant difference in the adjusted proportional changes in the levels of mean 2,4-DCP as indicated by BMI (*p* = 0.025) after necessary adjustments were made for potential covariates. However, the concentrations were not significantly correlated with the demographic factors, such as age, sex, level of education, household income, smoking status, or geographical residence.

From Table 4, the population-weighted geometric mean concentration of 2,5-DCP in urine samples was 0.44 μg/L at 95% (CI = 0.41–0.48). There was no significant relationship between the mean concentrations and BMI, age, sex, level of education, household income, cigarette smoking, or the geographical residence of the participants. Furthermore, adjusted proportional changes in levels did not significantly differ with demographic factors, with the exception of the place of residence (*p* = 0.045).

## 4. Discussion

In the current study, 2,4-DCP was detected in 63.4% of participants, while 2,5-DCP was detected in 97.9% of participants. These results indicate that there is a wide exposure to these chemicals in the Korean adult population. The geometric mean level of 2,4-DCP concentration in the urine of Korean adults was 0.14 μg/L. According to the US National Health and Nutrition Examination Survey (NHANES) that was conducted between 2003 and 2010, a value of 0.88 μg/L was reported in the population aged 20–59 years [22]. A study conducted in Germany reported a level of 0.54 μg/L in Germans aged 18–69 years [23]. Given that the levels of 2,4-DCP do not significantly vary with age, the body burden of 2,4-DCP is assumed to be lower in the Korean population compared to the U.S. or German populations.

The geometric mean concentration level of 2,5-DCP in the urine of Korean adults was 0.44 μg/L. This value is markedly low compared to that obtained in the U.S. population. According to the NHANES 2007–2010, a concentration of 6.05 μg/L was reported in U.S. adults [11]. The German Environmental Survey reported a level of 1.85 μg/L [23]. Both levels are way higher than that reported in the current study. Dichlorophenols remain in use in many countries worldwide. Some countries have attempted to decrease the production and import of these chemicals and their precursors [22]. Therefore, the different body burdens of 2,4-DCP and 2,5-DCP among the Korean, U.S., and German populations may be attributable to differences in exposure levels. In the NHANES, dichlorophenol concentrations were reported to be significantly lower in non-Hispanic whites compared to Hispanics or non-Hispanic blacks [11,22]. Therefore, a person’s ethnicity may also be related to the differences in urinary concentrations of dichlorophenols among populations.

Demographic factors, including place of residence and smoking, are reportedly related to urinary levels of some phenols and phthalates, although the causal nature of these associations is unclear. The urinary levels of mono-benzyl phthalate (MBzP) and mono-n-butyl phthalate (MnBP) were higher in the rural area (MBzP *p* < 0.001; MnBP *p* = 0.002) than in the urban area [24]. Additionally, smoking has been reported to significantly increase the level of bisphenol A and to decrease the level of triclosan (*p* < 0.05) [25,26]. In addition, smokers showed a significantly higher excretion of o-cresol (median: 23 vs. 33 μg/L), m-cresol (median: 43 vs. 129 μg/L), and 4-ethylphenol (median: 25 vs. 124 μg/L) [27]. However, there are no studies that have reported associations between specific demographic factors and urinary concentrations of 2,4-DCP and 2,5-DCP. In the current study, the adjusted proportional changes indicated that there was a significant correlation between BMI and levels of 2,4-DCP in the urine. In addition, subjects who resided in rural areas reported a higher level of 2,5-DCP concentration, as opposed to the subjects who resided in urban areas. The geographical place of residence and obesity influenced the level of concentration levels of 2,4-DCP and 2,5-DCP, suggesting that variations in the concentration of these chemicals was possibly influenced by place of residence and food intake.

This is the first study assessing the urinary concentrations of 2,4-DCP and 2,5-DCP in Korean adults using nationally representative data. The strength of this study is that it was possible to obtain concentration levels of 2,4-DCP and 2,5-DCP in the urine samples of participants, and establish how these concentrations were related to different demographic factors among adults from Korea using nationally representative data. Furthermore, urinary concentrations of these chemicals in the population were assessed using sample weights, which may have increased the accuracy of the national data. One limitation of this study was the use of a single spot-urine sample. There is a possibility that there was a variation in the concentration of the 2,4-DCP and 2,5-DCP within a person over a variation in time. However, the results of the studies on the urinary 2,4-DCP and 2,5-DCP concentrations by time of day of urine collection [28] or by visit of sample collection in a cohort study [29] showed that there was no significant temporal (intra-day or inter-day) variability of urinary 2,4-DCP and 2,5-DCP concentrations. In addition, it is known that approximation of mean population levels by use of a single spot sample for each participant is a more reliable approach in cross-sectional studies [15]. Future studies should focus on identifying sources of exposure to these toxic chemicals and assess the potential effects on human health.

## 5. Conclusions

A considerable percentage of the Korean adults, aged 18–69 years old, have urinary levels of 2,4-DCP and 2,5-DCP above 0.05 μg/L. The geometric mean levels were 0.14 μg/L (95% CI = 0.13–0.16) and 0.44 μg/L (95% CI = 0.41–0.48), respectively. Among the demographic characteristics studied, BMI and place of residence were significantly associated with the adjusted proportional changes in 2,4-DCP and 2,5-DCP levels, respectively. These findings suggest the need for policies to reduce exposure to these chemicals to prevent adverse effects in high-risk groups.

## Figures and Tables

**Table 1:** Selected concentration percentiles of 2,4-dichlorophenol in the urine collected from the Korean population aged between 18 and 69 years by sex and age sub-groups.

Variable	*N*	% > LOD *	Percentile ^1^					Max
			50th		75th	90th	95th	
Total	1865	63.4	0.08 (0.11)		0.56 (0.58)	1.66 (1.75)	3.32 (4.11)	83.6 (249.5)
Gender								
Men	802	63.1	0.09 (0.10)		0.58 (0.48)	1.90 (1.51)	3.52 (3.60)	72.0 (34.5)
Women	1063	63.7	0.08 (0.13)		0.55 (0.64)	1.48 (1.85)	2.97 (4.20)	83.6 (249.5)
Age (years)								
<30	247	62.8	0.08 (0.09)		0.56 (0.50)	2.53 (1.77)	4.26 (4.71)	58.4 (65.2)
30–39	412	64.1	0.08 (0.12)		0.52 (0.48)	1.54 (1.40)	3.23 (3.27)	72.0 (37.3)
40–49	454	63.9	0.09 (0.12)		0.59 (0.58)	1.71 (2.13)	4.48 (4.77)	62.8 (155.9)
50–59	430	63.5	0.16 (0.17)		0.68 (0.70)	1.70 (1.78)	3.30 (4.06)	83.6 (249.5)
≥60	322	62.4	0.08 (0.10)		0.48 (0.61)	1.30 (1.40)	2.20 (2.74)	21.0 (62.1)

* Limit of detection (LOD) = 0.05 μg/L. ^1^ The expression of percentiles is based on the volume concentrations of samples (μg/L), and concentrations adjusted for creatinine (μg/g creatinine) are shown in parentheses.

**Table 2:** Selected concentration percentiles of 2,5-dichlorophenol in the urine collected from the Korean population aged between 18 and 69 years by sex and age sub-groups.

Variable	*N*	% > LOD *	Percentile ^1^					Max
			25th	50th	75th	90th	95th	
Total	1865	97.9	0.11 (0.13)	0.40 (0.38)	0.98 (1.05)	3.20 (3.69)	6.70 (7.43)	63.0 (100.2)
Gender								
Men	802	98.0	0.12 (0.11)	0.42 (0.32)	0.97 (0.86)	3.04 (3.02)	6.96 (6.14)	49.1 (41.9)
Women	1063	97.8	0.10 (0.15)	0.39 (0.44)	0.99 (1.19)	3.30 (4.22)	6.50 (8.04)	63.0 (100.2)
Age (years)								
<30	247	98.4	0.17 (0.11)	0.44 (0.38)	1.14 (0.94)	3.02 (2.47)	6.36 (6.44)	46.3 (50.8)
30–39	412	98.5	0.16 (0.15)	0.43 (0.41)	0.98 (1.09)	3.25 (3.94)	7.53 (6.46)	38.2 (40.9)
40–49	454	98.2	0.08 (0.14)	0.41 (0.40)	0.97 (1.11)	3.20 (3.43)	6.00 (6.32)	34.0 (100.2)
50–59	430	97.4	0.08 (0.12)	0.35 (0.33)	0.92 (0.98)	3.70 (4.21)	8.20 (9.72)	23.0 (36.1)
≥60	322	96.9	0.08 (0.12)	0.37 (0.40)	1.00 (1.08)	2.90 (3.06)	5.70 (6.51)	63.0 (79.3)

* LOD = 0.05 μg/L. ^1^ The expression of percentiles is based on the volume concentrations of samples (μg/L), and concentrations adjusted for creatinine (μg/g creatinine) are shown in parentheses.

**Table 3:** Population-weighted geometric means and adjusted proportional changes in concentrations of 2,4-DCP with regard to demographic factors in the Korean adult population aged between 18 and 69 years.

Variable	*N*	Geometric Mean (95% CI), μg/L	*p*-Value ^a^	Adjusted Proportional Change (95% CI) ^b^	*p*-Value
Total		0.14 (0.13–0.16)		–	
Gender					
Men	802	0.14 (0.12–0.16)	0.400	1.01 (0.76–1.34)	0.960
Women	1063	0.15 (0.13–0.17)		1.00 (reference)	
Age (years)					
<30	247	0.16 (0.12–0.21)	0.784	1.00 (reference)	0.263
30–39	412	0.12 (0.10–0.15)		0.80 (0.58–1.11)	
40–49	454	0.13 (0.11–0.16)		0.90 (0.64–1.27)	
50–59	430	0.16 (0.14–0.20)		1.08 (0.74–1.58)	
≥60	322	0.13 (0.11–0.16)		0.89 (0.59–1.33)	
Body mass index					
<18.5	56	0.16 (0.08–0.32)	0.296	1.00 (reference)	0.025
18.5–22.9	816	0.16 (0.14–0.19)		1.03 (0.51–2.08)	
23.0–24.9	453	0.14 (0.12–0.17)		0.89 (0.44–1.80)	
≥25.0	540	0.11 (0.10–0.14)		0.72 (0.36–1.46)	
Educational level					
<High school	529	0.16 (0.13–0.19)	0.533	1.00 (reference)	0.416
High school	705	0.13 (0.11–0.15)		0.83 (0.64–1.10)	
>High school	631	0.15 (0.12–0.17)		0.88 (0.63–1.24)	
Average income (US$/month)					
<900	399	0.13 (0.11–0.16)	0.944	1.00 (reference)	0.652
900–2699	894	0.15 (0.13–0.17)		1.19 (0.91–1.57)	
2700–4500	423	0.14 (0.11–0.17)		1.14 (0.82–1.58)	
>4500	149	0.14 (0.10–0.20)		1.10 (0.72–1.68)	
Cigarette smoking					
Never	1232	0.15 (0.13–0.17)	0.863	1.00 (reference)	0.132
Former	228	0.11 (0.09–0.15)		0.79 (0.56–1.11)	
Current	405	0.15 (0.12–0.18)		1.09 (0.79–1.49)	
Residential location					
Rural	448	0.15 (0.13–0.18)	0.468	1.10 (0.88–1.37)	0.389
Urban	1417	0.14 (0.13–0.16)		1.00 (reference)	

^a^ *p* is determined by either linear trend test or survey *t*-test. ^b^ The exponentiated *β*-coefficient from a multiple regression of log-linear that included all covariates and creatinine concentrations in the urine samples.

**Table 4:** Population-weighted geometric means and adjusted proportional changes in concentrations of 2,5-DCP with regard to demographic factors in the Korean adult population aged between 18 and 69 years.

Variable	*N*	Geometric Mean (95% CI), μg/L	*p*-Value ^a^	Adjusted Proportional Change (95% CI) ^b^	*p*-Value
Total		0.44 (0.41–0.48)		–	
Gender					
Men	802	0.44 (0.39–0.49)	0.871	1.07 (0.86–1.33)	0.547
Women	1063	0.44 (0.40–0.49)		1.00 (reference)	
Age (years)					
<30	247	0.48 (0.40–0.59)	0.087	1.00 (reference)	0.365
30–39	412	0.45 (0.39–0.53)		0.88 (0.68–1.14)	
40–49	454	0.42 (0.36–0.49)		0.79 (0.60–1.03)	
50–59	430	0.41 (0.35–0.48)		0.76 (0.56–1.04)	
≥60	322	0.40 (0.33–0.47)		0.73 (0.53–1.01)	
Body mass index					
<18.5	56	0.33 (0.21–0.53)	0.247	1.00 (reference)	0.432
18.5–22.9	816	0.45 (0.40–0.51)		1.38 (0.86–2.23)	
23.0–24.9	453	0.42 (0.36–0.49)		1.33 (0.82–2.16)	
≥25.0	540	0.45 (0.39–0.53)		1.46 (0.90–2.37)	
Educational level					
<High school	529	0.41 (0.36–0.47)	0.422	1.00 (reference)	0.560
High school	705	0.45 (0.40–0.52)		1.04 (0.83–1.30)	
>High school	631	0.44 (0.39–0.50)		0.94 (0.71–1.23)	
Average income (US$/month)					
<900	399	0.43 (0.36–0.51)	0.993	1.00 (reference)	0.939
900–2699	894	0.44 (0.39–0.49)		1.00 (0.80–1.26)	
2700–4500	423	0.46 (0.39–0.55)		1.07 (0.80–1.41)	
>4500	149	0.41 (0.32–0.54)		0.99 (0.70–1.40)	
Cigarette smoking					
Never	1232	0.45 (0.40–0.49)	0.569	1.00 (reference)	0.730
Former	228	0.41 (0.34–0.51)		0.90 (0.68–1.18)	
Current	405	0.44 (0.38–0.52)		0.96 (0.75–1.22)	
Residential location					
Rural	448	0.46 (0.40–0.54)	0.461	1.09 (0.90–1.30)	0.045
Urban	1417	0.43 (0.40–0.47)		1.00 (reference)	

^a^ *p* is determined by either linear trend test or survey *t*-test. ^b^ Exponentiated *β*-coefficient from a multiple regression of log-linear that included all covariates and creatinine concentrations in the urine samples.
